# Case report: intravascular ultrasound-guided entry to an anomalous highly angulated circumflex coronary artery originating from the right sinus of Valsalva

**DOI:** 10.1093/ehjcr/ytae363

**Published:** 2024-08-20

**Authors:** Zhihong Yao, Afshin Khalatbari, Aleem Khand

**Affiliations:** Department of Cardiology, Liverpool Heart and Chest Hospital, Thomas Drive, Liverpool L14 3PE, UK; Department of Cardiology, Liverpool Heart and Chest Hospital, Thomas Drive, Liverpool L14 3PE, UK; Department of Cardiology, Liverpool Heart and Chest Hospital, Thomas Drive, Liverpool L14 3PE, UK

**Keywords:** IVUS, PCI, CTCA, Anomalous coronary artery, Case report

## Abstract

**Background:**

Anomalous coronary arteries originating from the contralateral sinus of Valsalva constitute a rare congenital anomaly. Most of such anomalous coronary arteries exhibit slit-like orifice that are often compounded by external compressive factors. Consequently, percutaneous coronary intervention (PCI) of these vessels poses considerable challenges, both in terms of cannulation but also intervention in often acutely angulated vessels.

**Case summary:**

A 61-year-old man, with a history of previous coronary artery bypass graft surgery and PCI presented with a history consistent with unstable angina. Notably, the left circumflex artery (LCX) in this individual exhibited an anomalous origin. Due to unfavourable anatomy and ambiguous LCX ostium take-off, previous operators had elected PCI of the saphenous vein graft (SVG) anastomosed to the obtuse marginal branch. A computed tomography scan on this occasion confirmed occlusion of the SVG and defined precise origin of anomalous coronary artery. Real-time live intravascular ultrasound (IVUS) positioned in the ascending aorta, in the right sinus of Valsalva, allowed visualization of the origin and wiring of the anomalous circumflex coronary artery and also facilitated successful PCI.

**Discussion:**

As far as we are aware, this is the first description of IVUS assisted wiring of an anomalous coronary artery. Intravascular ultrasound also facilitated decision making in this complex angioplasty of an angulated and heavily diseased circumflex coronary artery.

**Learning points**

- Anomalous coronary arteries often have slit-like orifices with extremely angulated ostium origin. Engaging and performing subsequent percutaneous coronary intervention in these vessels can present as a significant technical challenge.
- Multi-imaging modalities such as computed tomography coronary angiogram and intravascular ultrasound are extremely useful in planning and performing percutaneous coronary intervention for anomalous coronary arteries with extremely angulated ostium origins.

## Introduction

Anomalous coronary arteries originating from the contralateral sinus of Valsalva constitute an uncommon congenital cardiac phenotype, observed in ∼0.26% of the general population.^1^ The right coronary artery (RCA) is most commonly identified anomalous artery originate from the contralateral sinus of Valsalva, followed by the left circumflex artery (LCX), and less frequently, the left anterior descending artery (LAD). Anomalous RCA and LAD can have intramural or inter-arterial course, which are associated with an increased risk of sudden cardiac death. In contrast, the anomalous LCX typically follows a retroaortic or retrocardiac course and is generally considered benign.^2^

Atherosclerosis affects anomalous arteries as frequent as those with a conventional origin and anatomy. However, percutaneous coronary intervention (PCI) is often considerably more challenging given anatomical origin, slit-like orifices, and anomalous course.^3,4^

We present a case of unstable angina in the context of known severe coronary artery disease, including an anomalous left circumflex coronary artery (LCX), in a patient with previous coronary artery bypass surgery (CABG).

## Summary figure

(*A*) Position the guide catheter into the right coronary artery (RCA) and advance a workhorse wire into the native RCA. (*B*) Retract the guide catheter to a neutral position within the right sinus of Valsalva, allowing the intravascular ultrasound (IVUS) catheter to precisely identify the origin of the anomalous left circumflex (LCX) ostium. (*C*) Use real-time IVUS guidance to navigate a second guidewire with a sharply angled tip towards the LCX ostium. (*D*) Successfully insert the guidewire into the anomalous LCX under IVUS guidance.

**Figure:**
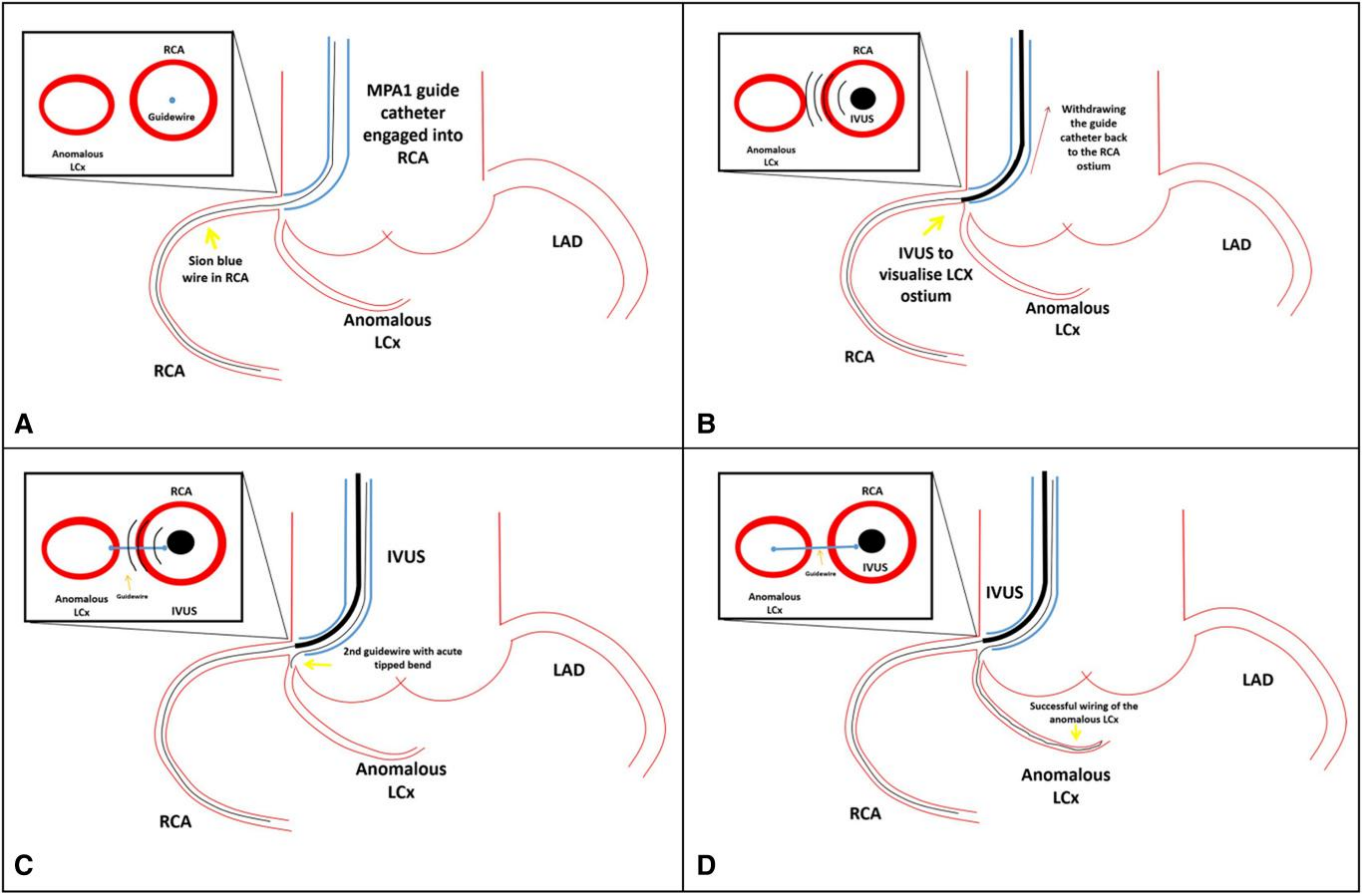


## Case presentation

A 61-year-old male, with an extensive cardiac history, was admitted with unstable angina. Thirteen years earlier, in 2009, he had undergone CABG. His grafts included a left internal mammary artery (LIMA) grafted to the LAD and a saphenous vein graft (SVG) to the principal obtuse marginal (OM) of the LCX. Notably, an anomalous LCX originating from the right sinus of Valsalva was identified at this time.

His past medical history also included a dual chamber pacemaker for conduction disease, antiphospholipid syndrome, hypertension, and hyperlipidaemia.

In 2012 and 2016, he represented with acute coronary syndrome. Critical stenosis of the SVG to OM was identified. On both occasions, PCI to the native diseased anomalous LCX was considered, but due to difficulties in cannulating this vessel, the strategy was changed to PCI of the SVG graft.

On physical examination, there was no abnormality identified in patient’s respiratory and cardiac functions. There were no peripheral signs of cardiac failure, and his physiological observations were within normal parameters.

**Figure 1:**
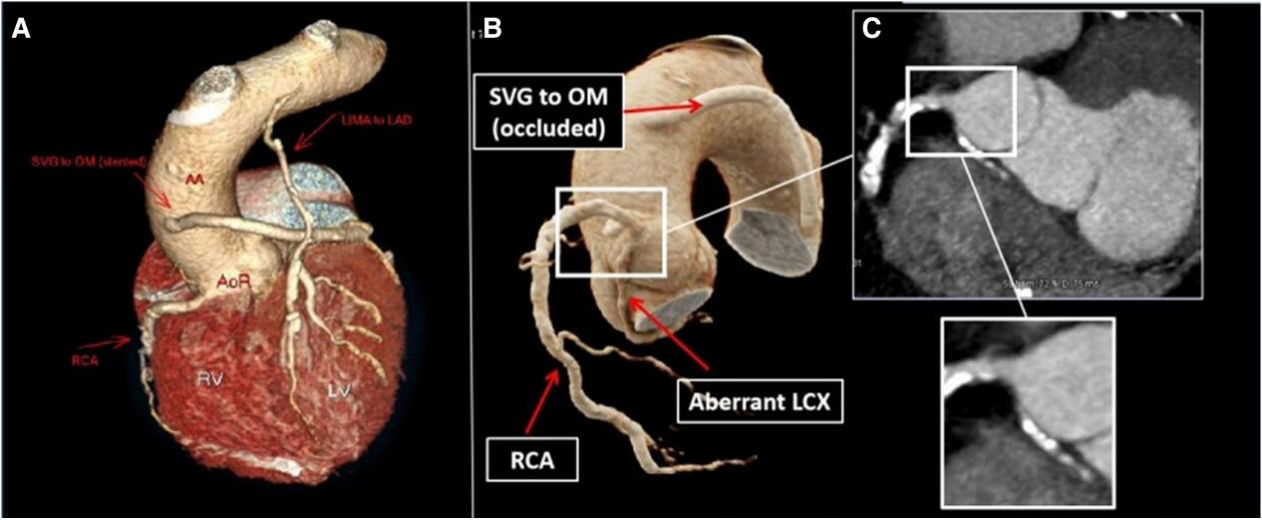
(*A*) Three-dimensional (3D) reconstructions of the patient’s native coronary arteries and bypass grafts are depicted using computed tomography coronary angiogram (CTCA). (*B*) 3D reconstruction demonstrating anomalous left circumflex (LCX) originates from the anterior/inferior position off the right coronary sinus of Valsalva, with an acute posterior-lateral angulation. (*C*) Two-dimensional (2D) coronal cross-section image showing severely calcified proximal anomalous LCX with an extremely inferior take-off from the right coronary sinus of Valsalva, 6 mm inferiorly to the right coronary artery (RCA) ostium.

An electrocardiogram exhibited sinus rhythm with infero-lateral T-wave inversion. High sensitive troponin T (Roche, Elecys) was 10 ng/L (99th percentile 14 ng/L). Echocardiography revealed preserved left ventricular systolic function. Computed tomography coronary angiography (CTCA) revealed a patent LIMA graft and a previously extensively stented but occluded SVG to OM branch. The CTCA also defined the anatomical origin and course of the anomalous LCX. Its origin was in the antero-inferior position of the right sinus of Valsalva, situated 6 mm inferiorly in relation to the RCA. It ran in an angulated posterolateral course (*Figure 1*). Invasive coronary angiogram studies of the native and grafts confirmed the CTCA findings. A pressure wire analysis of moderately diseased RCA revealed an instantaneous wave-free ratio (iFR) of 1.0. The anomalous LCX was visualized solely in a non-selective image on withdrawing the Judkin Right 4 (JR4) catheter (Mach 1™, Boston Scientific, USA) from the ostia of the RCA. Despite multiple catheters and projections of the X-ray equipment, efforts to thread the LCX with a guidewire proved futile.

His medical therapy was optimized but he remained very symptomatic with Canadian classification system (CCS) 3 angina. A stress cardiac magnetic resonance imaging demonstrated posterolateral ischaemia strongly suggesting the LCX as the culprit. Consequently, a decision was made to proceed with elective PCI to native LCX but on this occasion with preprocedural planning with CTCA and periprocedural use of intravascular ultrasound (IVUS).

**Figure 2:**
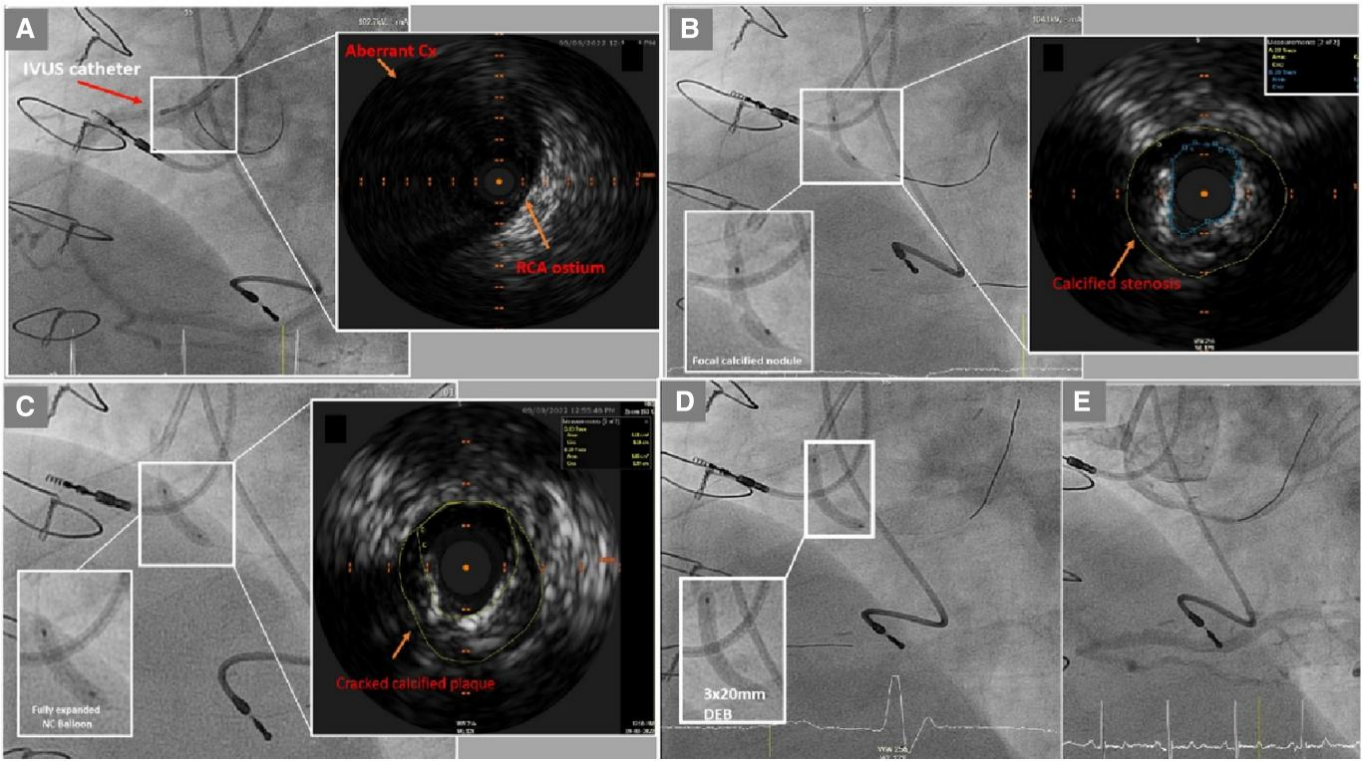
(*A*) Left anterior oblique (LAO) cine projection showing an intravascular ultrasound (IVUS) catheter placed at the RCA ostium in the right coronary sinus of Valsalva. The IVUS reveals the ostium of the anomalous LCX artery positioned at the 11 o’clock position relative to the RCA ostium. (*B*) LAO cine projection demonstrating an under-expanded 2.5 × 12 mm semi-compliant balloon with a focal calcified nodular lesion in the proximal anomalous LCX. The corresponding IVUS indicates severe calcified stenosis in the proximal anomalous LCX, with a minimum luminal area (MLA) of 3 mm² and a vessel area of 11 mm², reflecting a 73% reduction in luminal area. (*C*) LAO cine projection depicting a fully expanded 3.0 × 12 mm non-compliant balloon following the application of a 2.5 × 10 mm Coronary Cutting Balloon (Wolverine™, Boston Scientific). The IVUS shows an increased MLA of 6 mm² with cracked calcified plaque in the treated segment. (*D*) LAO cine projection demonstrating a well-expanded 3.0 mm × 20 mm drug-eluting balloon (SeQuent® Please NEO, B. Braun Melsungen AG, Germany) at 8 atmospheres (atm) within the proximal anomalous LCX. (*E*) Coronary angiogram in LAO projection showing a good final angiographic result following the application of the drug-eluting balloon therapy without any local complications.

The subsequent invasive angiogram was performed via the left radial artery. A 6 French Multipurpose-1 (MP1) guide catheter (Mach1™, Boston Scientific, USA) was used engage the RCA. A Sion blue wire (Asahi INTEC, Japan) was threaded into the native distal RCA, and subsequently, the guide catheter was repositioned from the RCA ostium back to the aorta. An IVUS catheter (Eagle Eye 20 MHz, Platinum Volcano, Philips, USA) was then carefully positioned just proximal to the RCA ostium, allowing visualization of the LCX ostium. Under the guidance of real-time IVUS visualization (*Figure 2A*), a second Sion blue guidewire (Asahi INTEC, Japan) featuring a 90° angulated tip was navigated from the right sinus of Valsalva to the ostium of the LCX. Through focused adjustments and precise steering of the guidewire under real-time IVUS visualization, operators successfully navigated and wire the anomalous LCX (Summary figure).

A 2.5 × 12 mm semi-compliant balloon (Emerge™, Boston Scientific, USA) was used to pre-dilate the proximal stenotic lesion. Cine image of balloon dilatation revealed under-expanded eccentric balloon with the suggestion of a focal calcified nodular lesion within the proximal LCX (*Figure 2B*). Following the pre-dilatation, IVUS confirmed a severely calcified stenosis with minimum lumen area (MLA) of 3 mm^2^. The actual normal segment of artery was 11 mm^2^ with media-to-media diameter measurement between 3.4 and 3.8 mm. This was substantially greater than the angiographic assessment reflecting a fore-shortened view of an acutely angulated artery.

The proximal calcified stenosis was subjected to successive balloon angioplasties using a 2.5 mm × 10 mm Coronary Cutting Balloon (Wolverine™, Boston Scientific, USA) followed by a 3.0 mm × 12 mm non-compliant balloon (NC Emerge™, Boston Scientific, USA) (*Figure 2C*). After these interventions, IVUS assessment demonstrated improved minimum luminal area (MLA 6 mm^2^), along with visible signs of fractured calcified plaque. A 3.0 × 20 mm drug-eluting balloon (SeQuent® Please NEO, B. Braun Melsungen AG, Germany) was applied over the diseased segment at 8 atmospheres (atm) for 60 s (*Figure 2D*). Drug-eluting stenting was not deployed due to an absence of good landing zones. Angiographic and IVUS result was acceptable (*Figure 2E*) (Supplementary material online). The patient had an uneventful recovery with improvement in his anginal symptoms. On 3 months follow-up, his angina symptom improved to CCS grade 1.

## Discussion

Coronary anomalies have been identified in 0.6–1.5% of general population.^5^ Anomalous origin of the LCX from the right sinus of Valsalva is one of the most common anatomical variations. PCI of anomalous coronary artery atherosclerotic diseases is associated with increased radiation exposure, contrast administration, and procedural duration.^2,3^

In this case, the infero-anterior origin of the LCX ostium in the right sinus of Valsalva and its angulated course rendered selective cannulation extremely challenging. The American College of Cardiology (ACC)/American Heart association (AHA) recommend coronary CT as Class I for a study of diagnostic anomalous aortic origin of coronary arteries.^6^ However, CTCA cannot currently provide real-time guidance capabilities during complex PCI. As demonstrated in our case, real-time IVUS positioned within the appropriate sinus of Valsalva can help direct cannulation of the anomalous artery.

Recent consensus guidelines from Europe and USA endorse the use of IVUS to address aorto-ostial ambiguity.^7^ Intravascular ultrasound proves invaluable in clarifying anatomical structures beyond the scope of angiography, especially in cases with an ambiguous position of the coronary ostium. A recent case series further emphasizes the practicality of real-time IVUS guidance in positioning ostial stents.^8^ Notably, this study concluded that real-time IVUS-guided ostium stent placement achieved a 100% rate of angiographic success, ensuring precise ostial stent deployment. In ambiguous ostial cases, placing the real-time IVUS catheter at the site of interest proves particularly useful in identifying the course and position of the vessel, thereby enhancing procedure accuracy for the operators. In our case, we positioned the IVUS catheter in the right sinus of Valsalva over the RCA guidewire to visualize the ambiguous LCX ostium. This helped the wiring of the anomalous LCX. A similar approach, utilizing real-time IVUS to navigate ambiguous coronary anatomy, has found application among chronic total occlusion (CTO) operators. In these instances, the IVUS catheter is strategically placed in a side branch, facilitating the visualization of the proximal cap of the central lumen of the CTO and aiding in the wiring technique.^8^ An additional advantage of IVUS in this context is the reduction in the use of contrast. This case contributes to the expanding utility of real-time IVUS, showcasing its diverse applications.

## Lead author biography


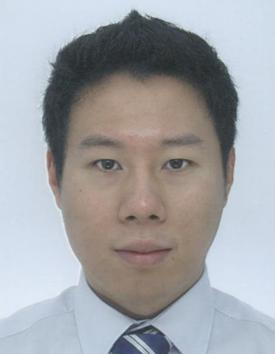


Final Year Interventional Cardiology Specialist Trainee.

## Supplementary Material

ytae363_Supplementary_Data

## Data Availability

The data that support the findings of this case report are available from the corresponding author upon reasonable request. Requests for data access should be directed to Al.K. at Aleem.Khand@lhch.nhs.uk. Additionally, some or all data may be available in Supplementary materials associated with this article on the *European Heart Journal – Case Reports* website. We encourage researchers interested in obtaining the data to contact the corresponding author and adhere to the data usage policies of the *European Heart Journal – Case Reports*.

## Supplementary material

**Consent:** The authors confirm that written, informed consent for submission and publication of this case report including images and associated text has been obtained from the patient in line with COPE guidance.

**Funding:** None to declare.
